# Effect of continuous professional development and clinical imaging guidelines on reducing inappropriate computerized tomography utilization among children and young patients in low resource settings: a before-and-after study

**DOI:** 10.4314/ahs.v25i3.21

**Published:** 2025-09

**Authors:** Harriet Nalubega Kisembo, Richard Malumba, Ezra Kato Nsereko, Deborah Babirye, Victoria Nakalanzi, Francis Xavier Kasujja, Elsie-Kiguli Malwadde, Elizeus Rutebemberwa, Simon Kasasa, Dina Husseiny Salama, Michael Grace Kawooya

**Affiliations:** 1 Department of Radiology, School of Medicine, College of Health Sciences, Kampala, Uganda; 2 Department of Radiology, Mulago National Referral and Teaching Hospital, Kampala, Uganda; 3 Department of Radiology, Ernest cook Ultrasound Research and Education Institute, Kampala, Uganda; 4 Department of Radiology, Entebbe Regional Referral Hospital, Entebbe, Uganda; 5 The Medical Research Council/Uganda Virus Research Institute, Entebbe, Uganda; 6 African Centre for Global Health and Social Transformation (ACHEST), Kampala, Uganda; 7 Department of Health Policy & Management, School of Public Health, College of Health Sciences Makerere University, Kampala, Uganda; 8 Department of Epidemiology & Biostatistics, School of Public Health, College of Health Sciences, Makerere University, Kampala, Uganda; 9 The National Center for Radiation Research and Technology (NCRRT), Egyptian Atomic Energy Authority (EAAEA), Cairo, Egypt

**Keywords:** Continuous professional Development, clinical imaging guidelines, computerized tomography utilization, young population, low resource settings

## Abstract

**Background:**

The overuse of CT examinations (CTEs), especially in low-resource settings (LRS), is a growing public health issue. Up to 50% of these CTEs are considered inappropriate, with children and young adults being particularly affected. While clinical imaging guidelines (CIGs) have been developed to address this issue, their effectiveness in LRS remains unclear.

**Objective:**

This study aimed to assess the impact of continuous professional development (CPD) and the introduction of CIGs on CT appropriateness in Ugandan hospitals.

**Methods:**

A before-and-after study was conducted in six public and private hospitals over 12 months.

**Results:**

Post-intervention, CTEs performed increased by 33%, with significant rises in public hospitals (30%) and private-for-profit hospitals (41%). Head CTs rose by 19%, and contrasted studies substantially increased by 252%. Conversely, trauma-related CTEs decreased by 8%. However, the overall proportion of inappropriate CTEs increased by 15%, with inappropriate contrasted examinations rising by 28% and non-trauma cases by 13%. Inappropriate non-contrasted CTEs and trauma-related CTEs reduced by 28% and 31%, respectively.

**Conclusion:**

The study highlights the challenges of consistently implementing CIGs in LRS, despite some improvements. It calls for more tailored interventions and further research to explore factors influencing guideline adoption to optimize CT utilization and improve patient outcomes.

## Introduction

Over the past two decades, Multi-Detector Computed Tomography (MDCT) has enhanced healthcare, particularly in emergencies and complex cases 1, 2. However, the growing use of CT imaging has raised concerns about its appropriateness, as it contributes significantly to global medical radiation exposure^3^, ^4^. According to United Nations Scientific Committee on the Effects of Atomic Radiation (UNSCEAR), CT scans are now the largest contributor to this radiation dose^5^. Alarmingly, 20%-50% of CT examinations (CTEs) globally are deemed inappropriate^6^, with 10%-20% occurring among children^7^-^9^.

In low resource settings (LRSs), inappropriate CT use is particularly problematic, resulting from overuse, misuse, or underuse. Overutilization often occurs when simpler; more cost-effective diagnostic methods would suffice, while underutilization may result from limited access to equipment or expertise^10^.

In sub-Saharan Africa, where the population is young and MDCT scanners are being rapidly acquired, cumulative radiation exposure is a growing public health concern.

Factors contributing to unnecessary CT use include limited knowledge among healthcare providers about radiation risks^11^-^13^, lack of adherence to referral criteria^14^, ^15^, and logistical challenges^16^.

Young patients are particularly vulnerable to radiation risks due to their longer life expectancy and heightened tissue sensitivity. Excessive CT scan use not only elevates the risk of radiation-induced cancers but also strains healthcare resources and increases costs^4^, ^17^-^19^

Evidence-based interventions like clinical imaging guidelines (CIGs), developed by organizations such as the American College of Radiology (ACR)^20^, the Royal College of Radiology (RCR)(21), and the World Health Organization (WHO)^22^, have proven effective in reducing inappropriate CTEs.

However, adapting these guidelines to LRSs is challenging given infrastructure limitations, financial constraints, disease pattern prevalence and variations in healthcare provider expertise and training. This study evaluates the effect of continuous professional development (CPD) and adapted CIGs on inappropriate CT utilization among young patients in six Ugandan hospitals.

## Methods

### Ethical approval

Ethical approval for the study was obtained from University's School of Medicine Research and Ethics Committee and the National Council for Science and Technology, with administrative clearance from all participating hospitals. A waiver of consent was granted to access patient records, and patient confidentiality was maintained by assigning unique identification numbers.

### Study Design

The study utilized a before-and-after design to assess the impact of CPD and CIGs on reducing inappropriate CT examinations (CTEs) among children and young patients.

### Study Setting

It was conducted in six purposively selected tertiary hospitals in Uganda, chosen for their referral status, teaching hospital affiliations, availability of CT services, and regional representation. The hospitals were categorized as public, private-for-profit (PFP), and private not-for-profit (PNFP).

At the time of the baseline study, public facilities had offered CT services for an average of over 15 years, PNFP centers for six years, and PFP institutions for eight years.

### Intervention

The intervention was designed following a baseline study that identified high rates of inappropriate CT examinations^23^. It involved conducting CPD sessions and implementing CIGs based on the European Society of Radiology iGuide^24^. Healthcare providers, including interns, medical officers, specialists, radiographers, radiologists, and administrative staff, were grouped together during routine hospital CPD sessions.

Senior radiologists and technologists delivered the CPD sessions, covering key topics such as radiation protection, justification and optimization of medical exposures, radiation doses (Table 1), and associated risks.

**Table 1:** Effective radiation doses for common diagnostic imaging modalities and equivalent number of chest X-rays doses

Diagnostic imaging modality	Effective dose (mSv)^a^	Equivalent number of chest X-rays resulting in the same effective dose	Following ranges are accepted (based on equivalent number of chest X-rays)
Chest X-ray	0.02	1	1
Cranial X-ray	0.07	3.5	0–10
Pelvic X-ray	0.7	35	10–50
Abdominal X-ray	0.7	35	10–50
Cranial CT	2.4	120	50–200
Chest CT	7.8	390	200–500
Abdominal CT	12	600	>500
Pelvic CT	10.5	525	>500
Abdominal US	0	0	0
Abdominal MRI	0	0	0

a Effective dose, health care level II countries, United Nations Scientific Committee on the Effects of Atomic Radiation (UNSCEAR) report 2008 (10)

**Figure 1:**
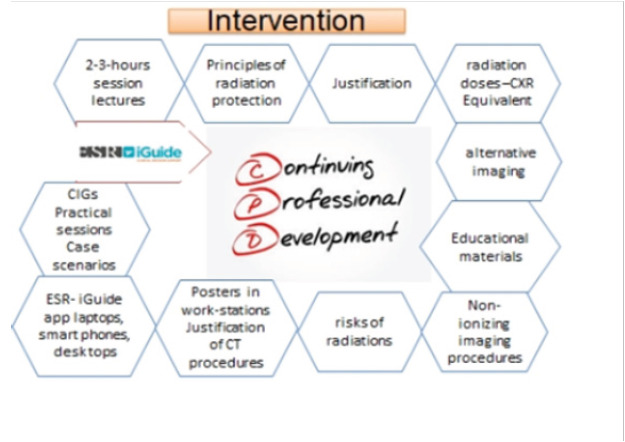
content of the intervention to reduce inappropriate CT requisitions

The sessions also emphasized alternative imaging modalities with less or no ionizing radiation. After the lecture, participants engaged in an interactive session where they accessed and downloaded the ESR-iGuide, clinical imaging guideline app. Educational materials, including International Atomic Energy Agency (IAEA) posters on radiation protection, were distributed to participants. The intervention content is summarized in the figure 1 below:

To reinforce learning, CPD sessions were repeated every 6 to 12 months, with each participating hospital receiving at least two sessions. However, Private Not-for-Profit (PNFP) hospitals had prior exposure to a similar intervention^25^. A pre- and post-intervention test was conducted to assess participants' knowledge of radiation safety and the justification of medical exposures. The results of these tests have been published separately^26^. The sessions contributed towards continuous professional development, a requirement for healthcare providers' license renewal.

The study tested the hypothesis that CIGs would reduce inappropriate CT requisitions by 10-15%. Data were collected retrospectively from CT request forms (CTRFs) for patients aged 35 years and below across six hospitals, focusing on head, chest, abdomen, spine, and trauma CTs. The analysis was conducted on eligible requests, with inappropriate requests categorized based on a two-grade scale derived from a computerized decision support (CDS) tool, the ESR-ACR iGuide. CTRFs were rated for appropriateness (score 7-9), score 1-6 and those with insufficient clinical information resulting in categorization as inappropriate.

Statistical analysis compared the number of inappropriate CT scans before and after the intervention using Pearson's chi-square test or Fisher's exact test, with significance set at P < 0.05. Data were analyzed by hospital type, patient demographics, anatomical region, and CT scan indication.

## Results

During the study, 2,119 CT examinations were conducted on patients aged 35 or younger across six hospitals, with 38% of the patients being female and 23% being children under 18 years. Table 2 below summarizes the total numbers and variations in different types of CT examinations performed on patients aged 35 or younger in 2018 and 2020 at the six participating hospitals.

**Table 2:** Total number (N) and percentage change (%) of CT examinations conducted on patients aged 35 years or younger before and after the intervention across the six participating hospitals

	Baseline	Post-intervention (N (%)	% Change	P-values	95% Confidence	Intervals
	909 (43)	1,210 (57)	14.2	<0.001	(0.0994, 0.1846)	
**Hospital Category**						
Public Hospital	300 (33)	608 (50)	17.25	<0.001	(0.1061, 0.2389)	
Private for Hospital	91(10)	238 (20)	9.66	0.037	(0.0169, 0.1763)	
Private not for profit	518 (57)	364 (30)	-26.91	<0.001	(-0.3326, -0.2056)	
**Gender**						
Male	572 (63)	741(61)	1.69	0.532	(-0.0698, 0.0360)	
Female	331 (37))	468 (39)	2.19	0.529	(-0.0462, 0.900)	
Missing	6 (10)	1 (0)	-0.58			
**Age categories 1**						
< 1	36 (4)	61(5)	1.08	0.807	(-0.0733, 0.0949)	
1 <= 5	63 (7)	80 (7)	0.32	0.940	(-0.0862, 0.0798)	
6 <= 10	48 (5)	66 (6)	0.17	0.968	(-0.0820, 0.0854)	
11 <= 15	52 (6)	55 (5)	-1.17	0.784	(-0.0955, 0.0721)	
16 <= 20	115(13)	161(13)	0.66	0.872	(-0.0737, 0.0869)	
Above 20	595(66)	786 (65)	-0.5	0.847	(-0.0557, 0.6928)	
Missing	0	1 (0)	0.08			
**Age categories 2**						
<18	232 (26)	307 (25)	-0.15	0.968	(-0.0758, 0.0728)	
≥18	677 (75)	902 (75)	0.07	0.975	(-0.0427, 0.0441)	
Missing	0 (0)	1 (0)	0			
**Anatomic Region**						
None^#^	34 (4)	2 (0)	-3.57	0.791	(-0.1213, 0.0499)	
Head	746 (82)	890 (74)	-8.52	<0.001	(-0.1252, -0.0452)	
Spine	16(2)	61 (5)	3.28	0.567	(-0.0518, 0.1174)	
Abdomen	48 (5)	101 (8)	3.07	0.502	(-0.0552, 0.1138)	
Chest	34 (4)	134 (11)	7.33	0.195	(-0.0097, 0.1563)	
PNS	20 (2)	12 (1)	-1.21	0.800	(-0.0974, 0.0732)	
Others	11 (1)	10 (1)	-0.38	0.931	(-0.0895, 0.0819)	
**Use of Contrast Media**						
Not contrasted	796 (88)	800(66)	-21.45	0.931	(-0.0895, 0.0819)	
Contrasted	113(12)	410(34)	21.45	<0.001	(0.1383, 0.2907)	
**Trauma-relate indication**						
Non-trauma	412 (45)	702 (58)	12.7	<0.001	(0.0666, 0.1874)	
Trauma	497 (55)	458 (38)	-16.83	<0.001	(-0.2307, -0.1059)	
Missing	0 (0)	50 (4)	4.13			

None:

# refers to those CT request forms with no anatomical region to be scanned

Table 3 presents a detailed comparison of the number and percentage differences in inappropriate CT examinations performed on young patients across various categories between 2018 and 2020.

**Table 3:** The number (n), proportion (%) and the difference in proportions of inappropriate CT examinations before and after intervention for patients aged ≤ 35 years

Inappropriateness	Baseline	Post-intervention	% change	p-value	95% CI	Column1
Variable	347 (38)	645 (53)	15.14	<0.001	(0.2382, 0.3626)	
**Gender**						
Male	139 (40)	**287 (44)**	4.44	0.386	(-0.5530, 0.1441)	
Female	207 (60)	358 (56)	-4.15	0.337	(10.1259, 0.4286)	
**Age categories 1**						
< 1	13 (4)	56(9)	4.93	0.549	(-0.0776, 0.1762)	
1 <= 5	27 (8)	45 (7)	-0.8	0.899	(-0.1335, 0.1175)	
6 <= 10	24 (7)	37 (6)	-1.18	0.852	(-0.1380, 0.1144)	
11 <= 15	26 (8)	37 (6)	-1.75	0.781	(-0.1434, 0.1084)	
16 <= 20	52 (15)	92 (14)	14.26	0.905	(-0.1278, 0.1132)	
Above 20	205 (59)	377 (59)	-0.63	0.883	(-0.0900, 0.0774)	
**Age categories 2**						
<18	105 (30)	200 (31)	0.75	0.893	(-0.1013, 0.1163)	
≥18	242 (70)	444 (69)	-0.9	0.807	(-0.0816, 0.0632)	
**Anatomic Region**						
None^#^	34 (10)	2 (0.3)	-9.49	0.653	(-0.2211, 0.0313)	
Head	239 (69)	412 (64)	-5	0.195	(-0.1248, 0.2481)	
spine	7 (2)	38 (6)	3.87	0.674	(-0.0896, 0.1670)	
Abdomen	27 (8)	92 (14)	6.48	0.375	(-0.0589, 0.1887)	
Chest	25 (7)	87 (14)	6.29	0.395	(-0.0613, 0.1871)	
PNS	8 (2)	7 (1)	-1.22	0.852	(-0.1416, 0.1172)	
Others	7 (2)	7 (1)	-0.93	0.888	(-0.1388, 0.1202)	
**Contrast Media Usage**						
Not contrasted	259 (75)	302 (47)	-27.82	<0.001	(0.3555, -0.2009)	
Contrasted	88 (25)	343 (53)	27.82	<0.001	(-0.1731, 0.3833)	
**Trauma Related indications**						
Non-trauma	228 (66)	507 (79)	12.89	<0.001	(0.0569, 0.2001)	
Trauma	119 (34)	88 (14)	-20.65	0.001	(-0.3179, -0.0951)	

NB; *p-value <0.05. NA = Assumptions for the normal approximation to the binomial were not met. The percentages shown above are for inappropriateness

# None: refers to those CT request forms with no anatomical region to be scanned

## Discussion

This study aimed to assess the impact of continuous professional development (CPD) and clinical imaging guidelines (CIGs) on reducing inappropriate CT examinations (CTEs) in a low-resource setting (LRS). Despite efforts to incorporate radiation protection and CIGs into training, the results showed a 33% overall increase in CT scans, accompanied by a 15% rise in inappropriate requests. These findings suggest that the intervention did not significantly improve CT utilization practices. The limited effect of a few educational sessions on behavioral change aligns with previous research on guideline implementation^27^, ^28^.

However, PNFP hospitals demonstrated a 27% decrease in inappropriate CT utilization, potentially due to prior exposure to similar interventions and higher consistency in attendance among practitioners. This finding is consistent with Forsetlund et al^29^, who noted that multi-session educational programs are generally more effective in supporting knowledge retention and behavior change than single-session efforts.

Overutilization of CT scans in private-for-profit healthcare facilities may stem from the direct financial benefits these institutions gain from increased utilization. In contrast, in public hospitals, the financial implications of overutilization are typically absorbed at higher administrative and management levels, rather than directly impacting the radiology department^30^-^32^.

This study found no clear influence of the duration of CT services or institutional maturity on decision-making processes, warranting further exploration. While public hospitals with longer experience may benefit from established workflows, better-trained staff, and greater oversight, they also face higher patient loads, which can contribute to overutilization due to resource constraints and demand pressures^33^. In contrast, PNFP and PFP hospitals with fewer years of experience may struggle with implementing proper utilization protocols, often due to limited expertise and weaker clinical governance structures.

Integrating evidence-based guidelines into clinical routines is challenging, as clinical behaviors are often resistant to change and require time to shift^34^, ^35^. Research indicates that behavior change is achievable but usually requires comprehensive, multi-level strategies targeting individual practitioners, healthcare teams, institutional practices, and the healthcare environment^27^, ^34^, ^35^. Effective interventions must be tailored to the specific context and population, addressing the unique characteristics of the guidelines as well as the barriers and facilitators relevant to their adoption^25^, ^36^, ^37^. No single implementation approach is universally effective; success depends heavily on adapting strategies to each setting^6^,^38^, ^39^.

Another possible reason for the limited impact is the absence of incentives to encourage change. Barriers such as limited access to alternative imaging modalities, knowledge gaps, a lack of monitoring and follow-up systems, absence of decision-support tools, high workloads, and understaffing may make behavior change seem unfeasible or unappealing^40^.

The increase in CT use could also be attributed to broader factors, including the global rise in cross-sectional imaging, advancements in CT technology, and greater public demand for diagnostic imaging^41^.

Additionally, guidelines developed for high-income countries (HICs) may be less effective in LRS without proper adaptation to local contexts and resources^25^, ^42^-^44^. The intervention might have also inadvertently raised provider awareness of CT availability, leading to an increase in requisitions, including inappropriate ones^45^.

Trauma-related procedures, which made up 45% of all CT examinations (CTEs), decreased by 20% post-intervention, along with a 16% reduction in inappropriate trauma-related scans. This decline may have been influenced by national lockdowns during the COVID-19 pandemic^46^. Traumatic brain injuries (TBIs) remain a key area for reducing unnecessary scans, as studies suggest that many could be avoided through clinical decision rules like the Canadian CT Head Rule^47^.

In contrast, inappropriate contrasted CTEs increased by 28%, likely due to limited awareness of guidelines or restricted access to non-ionizing imaging options such as ultrasound and MRI.^42^-^44^, ^48^-^50^. This limited access to alternatives can expose patients to avoidable risks

The increase in contrast-enhanced CT scans raises safety concerns due to potential adverse reactions, higher radiation exposure, and contrast-induced nephropathy (CIN), especially among vulnerable groups like young and elderly patients^51^. Contrast studies necessitate additional imaging, leading to increased radiation doses and a higher long-term cancer risk, while iodinated contrast agents can strain kidney function^51^, ^52^.

Additionally, the lack of radiologist consultation before ordering CTs has contributed to inappropriate usage, as radiologists play a key role in ensuring exposures are clinically justified (53-55). Studies indicate that imaging referrers tend to exercise greater caution in ordering when they know requests may be reviewed and potentially denied^56^.

### Limitations

The study identifies several limitations that may have affected the intervention's impact, including secular trends, seasonal effects, and random fluctuations, highlighting the importance of robust experimental designs for future research. Constraints such as a limited number of CPD sessions, a short follow-up period, and the inability to measure individual intervention components also limited the findings. Nonetheless, the multi-center design improves the generalizability of results across hospitals in Uganda, though baseline limitations remain relevant to this analysis^23^.

## Conclusion

Despite the well-designed intervention, the study did not observe a statistically significant reduction in inappropriate CT scans, highlighting the challenges of implementing clinical guidelines in LRS. More comprehensive, context-sensitive strategies and imaging protocols are needed to improve CT scan appropriateness and patient care.

Future research should focus on longer follow-up periods, varied educational approaches such as continuous medical education, decision-support tools, audits with feedback, interdisciplinary collaboration, and tailored training, qualitative studies on healthcare providers' adherence to guidelines, and cost-effectiveness analysis to improve CT referral practices and inform policymaking.
